# Microfluidic Spontaneous Emulsification for Generation of O/W Nanoemulsions—Opportunity for In‐Space Manufacturing

**DOI:** 10.1002/adhm.202203363

**Published:** 2023-05-04

**Authors:** Svenja Schmidt, Anh The Nguyen, Huy Quang Vu, Nam Nghiep Tran, Maria Sareela, Ian Fisk, Volker Hessel

**Affiliations:** ^1^ School of Chemical Engineering The University of Adelaide Adelaide 5005 Australia; ^2^ Andy Thomas Centre for Space Resources Adelaide 5005 Australia; ^3^ International Flavour Research Centre Division of Food Nutrition and Dietetics University of Nottingham Sutton Bonington Campus Loughborough LE12 5RD UK; ^4^ South Australian Research and Development Institute SARDI Adelaide 5064 Australia; ^5^ International Flavour Research Centre (Adelaide) School of Agriculture Food and Wine and Waite Research Institute The University of Adelaide PMB 1 Glen Osmond South Australia 5064 Australia

**Keywords:** continuous processing, flow chemistry, nanoemusions, nanoparticles, space manufacturing

## Abstract

The use of microfluidics for oil‐in‐water (O/W) nanoemulsification via spontaneous self‐assembly is demonstrated. As this is known to be a longish process, both single‐ and multicontact microfluidic reactors are tested, the latter providing a longsome, constant microfluidic treatment to maintain advanced phase and interfacial mass transfer. Microfluidic devices provide strong advantages above conventional systems for spontaneous emulsification, with droplet sizes of 62 nm at desired surfactant‐to‐oil ratios (SOR) and a decrease of 90% in process time. Multicontact microfluidics have better performance than their single‐contact counterparts, while critical aspects, e.g., process robustness, are also discussed. Ternary phase diagram analysis of the three components (oil, water, surfactant) allow to decide for the right mixing ratio and sequence of mixing steps for the nanoemulsions. Microfluidic spontaneous emulsification meets objective functions of the intended application to provide fortified beverages to astronauts in space exploration. In that viewpoint, an advantage is to achieve stable nanoemulsions at a level of concentrations much higher as compared to application (human intake), allowing a dilution factor to the final product of up to 100. This decreases notably the process time and allows for process flexibility, e.g., to dilute or tailor Earth‐prepared nanoemulsion concentrate payloads in space.

## Graphical abstract

The world's leading space agencies are currently working on ways to sustain astronauts for long‐term space missions, as current space foods might contribute to so‐called space anorexia. Thus, it is proposing to combine self‐assembly surfactant systems (solvent‐free spontaneous emulsification) with microfluidics to reliably obtain beverage nanoemulsions, which can be employed to produce fortified beverages, thereby improving health and well‐being in space.

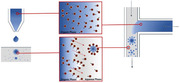

## 1 Introduction

Nanoemulsions are used for drug delivery, food, cosmetics, pharmaceuticals, and material synthesis,^[^ ^1^, ^2^, ^3^, ^4^ ^]^ but can also serve as a model system to understand nanoscale colloidal dispersions. Applications are not restricted to Earth alone. In‐space manufacturing, i.e., currently on a spacecraft as the International Space Station (ISS) and possibly in future on space habitats on the moon, offers promises for processing colloidal systems due to the lack of gravity. Instrumental advantages are the perfect spherical shape of droplets and allowing to study mass transfer in an ideal environment, i.e., the lack of gravitational forces. Nanoemulsified fortified food items and pharmaceuticals offer diverse advantages for space explorers and astronauts, ranging from high product stability to high bioavailability. These products can be either made on Earth and transported to space or made on the spot by in‐space manufacturing. The latter has the advantage of producing a personalized product, i.e., a medicine based on accumulative exposure data and real‐time feedback from health monitoring sensors. Our laboratory, for example, is targeting a benchtop beverage dispenser that allows a personalized beverage formulation (i.e., product with bespoke nutritional profiles and quality profiles including individualized flavor systems) that can be applied in remote places, in particular space, to increase health and well‐being.^[^ ^5^ ^]^ The dispenser may also produce sauces, as sauces contain fat levels that are comparable to this system.

A nanoemulsion is a specific nanostructure consisting of liquid droplets (i.e., dispersed phase) dispersed in another liquid (i.e., continuous phase) of a different polarity, which are otherwise immiscible.^[^ ^1^, ^2^, ^3^, ^4^ ^]^ The mixture is stabilized by surface‐active molecules called emulsifiers which determine the degree of dispersion of the two phases. Typical nanoemulsions are oil‐in‐water (O/W) nanoemulsions and water‐in‐oil (W/O) nanoemulsions. Compared to conventional emulsions, the mean droplet diameter of nanoemulsions is considerably smaller, and usually defined as below 200 nm.^[^ ^6^ ^]^ Due to the droplets’ small size, nanoemulsions are optically transparent or slightly turbid, and less susceptible to instability mechanisms induced by gravitation. O/W nanoemulsions have gained interest in pharmaceutical and food research, as they are elemental constituents of many foods and can encapsulate hydrophobic active pharmaceutical ingredients or hydrophobic nutraceuticals and flavor agents, respectively. These compounds are often sensible to environmental conditions; thus, encapsulation can protect bioactive molecules from untimely degradation while the nanostructure promotes bioavailability.

Typically, nanoemulsions are produced by high‐energy emulsification processes, such as microfluidizers, high‐pressure valve homogenizers, and ultrasound homogenizers. These devices apply strong mechanical forces to induce droplet formation to the bulk oil phase. Yet, they require a high external energy input, while generating excessive heat during the emulsification, which can damage encapsulated temperature‐sensitive molecules.^[^ ^7^ ^]^ As counterpart, low‐energy emulsification processes employ intermolecular interactions to promote emulsion formulation, such as emulsion phase inversion and spontaneous emulsification, and thus, prevent the formation of temperature peaks in the emulsions.^[^ ^8^, ^9^ ^]^ We propose to employ the spontaneous emulsification as an alternative to traditional emulsification technologies, particularly for food‐grade applications which require a low‐temperature nanoemulsification.

**Figure 1:**
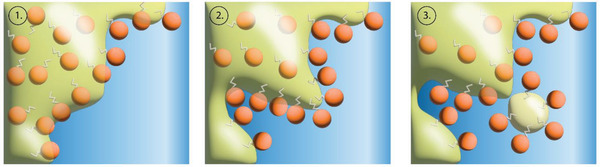
Lapse of spontaneous emulsification: Once the oil phase and aqueous phase come into contact, the surfactant moves to the interface, and through rapid diffusion forms droplets. Adapted with permission.^[^ ^10^ ^]^ Copyright 2015, Elsevier.

Spontaneous emulsification (SE) refers to the surfactant being initially dissolved in oil, and due to its hydrophilic nature moving from the oil phase to the aqueous phase at the oil‐water interface, whereas conventional emulsification (CE) refers to the surfactant being initially dissolved in the aqueous phase and stabilizing newly formed oil droplets by moving to the oil‐water‐interface. A surfactant, which is miscible in both aqueous phase and oil but is more hydrophilic than lipophilic, is mixed with the oil before being brought into contact with the aqueous phase.^[^ ^10^ ^]^ The surfactant then rapidly diffuses from the oil phase to the aqueous phase, thereby forming droplets at the interphase.^[^ ^11^ ^]^ While this approach is reported to be efficient at producing emulsions in the nanometer range, it requires high concentration of synthetic surfactants, which is not attractive for application in the food industry.^[^ ^10^ ^]^ We focus on a solvent‐free variant of the spontaneous emulsification, as lack of organic solvents is favorable for food‐grade applications (**Figure** **1**).

Therefore, we propose to combine the spontaneous emulsification with a continuous‐flow microfluidic process. Microfluidics studies utilize fluids on small scale. Channel diameters range typically between a few hundreds of micrometers up to a few millimeters, but can also be as small as tens of micrometers.^[^ ^12^, ^13^, ^14^ ^]^ On such a small scale, physical phenomena such as capillary forces and surface tension prevail which are otherwise negligible,^[^ ^15^ ^]^ and allow for precise control of the fluids. This provides both large interfacial area for interface mass transfer and strong convection within the phase for interface renewal. Microfluidic liquid/liquid contacting methods are known to reliably produce droplets of a uniform size, which is favorable for emulsion production as a narrow droplet size distribution increases stability. However, the diameter of microfluidic‐formed droplets usually do not go below a few micrometers.^[^ ^16^ ^]^

Our study is motivated by providing nutrients and medicines to astronauts in the context of long‐term space exploration. Fortified beverages can supplement fresh (in‐space produced) or packaged (taken as payload) food and medicine formulations; especially when concerning essential nutrients, needed in minute scale (micrograms) and with a strong personal variability of demand. The vision is an automated machine which can produce individualized (designer) beverages at the spot in a spacecraft or space habitat, supported by health monitored data and expert decision (e.g., telemedicine), following a forecast on the role of future “astro‐pharmacists” published.^[^ ^17^ ^]^ Conventional technologies usually need gravity, e.g., when stirred in a beaker, and will hardly work under microgravity and possibly have problems under reduced gravity. Microfluidic operation, often described as flow chemistry, is the only to date known method of chemical engineering that does not rely on gravity and therefore has been used on‐board ISS (besides macrofluidics).^[^ ^18^ ^]^ Microfluidics are considered as prime method for space chemistry,^[^ ^19^, ^20^ ^]^ and a “space chemistry” association has recently been propelled by the American Chemical Society, NASA, and other interest groups.^[^ ^21^ ^]^

**Figure 2:**
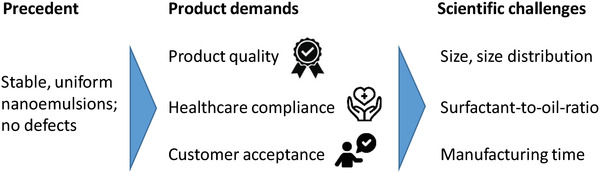
This study aims at the precedent (“proof of concept”) of demonstrating microfluidic spontaneous emulsification to meet product demands of fortified designer beverages. The latter requires to solve scientific challenges beyond the precedent.

In view of the future use of our technology for in‐space manufacturing, meaning to produce fortified designer beverages for astronauts, the condition precedent is to i) identify oil‐water‐surfactant compositions that support targeted beverage nanoemulsions (see **Figure** **2**). Key targets of a suited beverage emulsions are ii) the quality of the droplet dispersion (average size, size distribution), iii) speed of beverage formation to meet practical consumer needs, and surfactant‐to‐oil ratio (SOR) to meet healthcare compliance issues as surfactants can be irritant to humans.

In summary, there is a need for a gravity independent, low energy, low temperature unit process that can formulate nanoemulsions in situ. We have therefore trialled a unique microfluidic—spontaneous emulsification approach for the development of nanodroplets and subsequent personalization of nutrients and quality traits. To our best knowledge, this is investigated here first time. Two modes of microfluidic emulsification are used. Single‐contact processing in segmented flow generates in one step oil‐water droplets which instantly decompose to smaller fragments by SE. Without SE, those droplets would form uniformly in size and a segmented flow. In our case, SE overrules this and we are unable to see the initial droplets, as the SE process starts immediately upon contact. Multicontact microfluidic processing ongoingly reshapes the oil‐water dispersion, meaning a constant flow disturbance that has been called re‐entrance flow effect^[^ ^22^ ^]^ and found use in microreactor technology, including specialized devices^[^ ^23^ ^]^ and commercial devices (the Corning low flow reactor used here^[^ ^24^ ^]^).

## 2 Experimental Section

### 2.1 Materials

The same oil, emulsifier, and aqueous phase were used as emulsion model system to study its characteristics and its behavior in different mixing experimental setups, with varying component concentrations. For the oil component, ultrafiltered pharmaceutical grade medium‐chain triglycerides (MCT) was acquired from Vials Direct (Salamander Bay, NSW, Australia). As emulsifier, the nonionic surfactant Tween 80 (Polysorbate 80) was bought from Sigma‐Aldrich (Truganina, VIC, Australia). The aqueous phase consisted of highly diluted phosphate buffer (5 mm; pH 7.4 at 25 °C) using a 1.0 m phosphate buffer solution (Sigma‐Aldrich, Truganina, VIC, Australia) and ultrapure water (Millipore Milli‐Q).

### 2.2 Methods

#### 2.2.1 Ternary Phase Diagram Studies

A ternary phase diagram was created to investigate the model system's mixing characteristics in batch and to identify suitable compositions for the emulsification process. High pressure liquid chromatography (HPLC) sample vials (*V* = 2 mL, clear glass) were filled with 1 g of mixture containing the model system's three components (MCT, Tween80, phosphate buffer). The composition of the mixture changed in 10 wt% increments or reduction for each component from vial to vial, totaling 100 wt% or 1 g. Per ternary phase diagram 66 sample vials were prepared in that manner, ranging from 0 to 100 wt% per component (see Table S1 in the Supporting Information).

In total, three ternary phase diagrams were created to investigate the different ways of mixing the components. For the first diagram, all three components were given in the vial and mixed thoroughly. For the second diagram, phosphate buffer and Tween 80 were given first in the vial, mixed thoroughly, and then MCT was added to the mixture, and mixed thoroughly. For the third diagram, MCT and Tween 80 were given first in the vial, mixed thoroughly, and then phosphate buffer was added to the mixture, and mixed thoroughly. All filled vials were positioned upright, and were not moved for 7 days, after which the mixtures were visually exanimated. The mixtures were classified in one of four categories, namely 1) two layers, 2) stable emulsion, 3) gel, and 4) single clear phase.

#### 2.2.2 Preparation of the Oil Phase

Throughout the emulsification experiments, either MCT 1) or a mixture of MCT and Tween 80 2) were used as oil phase. In the latter case, the ratio between Tween 80 and MCT (surfactant‐to‐oil ratio, SOR) was varied to obtain different surfactant concentrations in the oil phase

(1) $$SOR=\frac{m_{s}}{m_{o}}$$

*m*_s_ and *m*_0_ describe the mass percentages of surfactant and oil, respectively. SORs ranged from 0.05 to 2.0, depending on the relevant experiment. The required amount of oil and surfactant were given in a beaker and thoroughly mixed at 600 rpm for at least 30 min (RCT Basic magnetic stirrer, IKA, Staufen, Germany) at room temperature.

#### 2.2.3 Density Measurement of the Oil Phase

The density of the oil phase (SORs: 0.05, 0.1, 0.25, 0.3, 0.35, 0.4, 0.45, 0.5, 0.6, 0.75, 1.0, 1.25, 1.5, 1.75, and 2.0) was determined by weighting a defined volume in an air‐conditioned room (room temperature: 22–24 °C). With a syringe (*V* = 10 mL, luer slip, concentric tip, Terumo (Philippines) Corporation, Laguna, Philippines) 10 mL of the oil phase was transferred to a beaker and weighted using a precision balance (PRseries, Ohaus, NJ). At low surfactant concentrations (SOR < 0.5), it was observed that the surfactant did not completely dissolve in the oil. Thus, in these cases the oil phase was stirred during sample removal to ensure an even distribution of both components. This step was repeated at least 10 times and the average weight, average density, and standard deviation was calculated. The density of MCT was determined using the same method.

#### 2.2.4 Viscosity Measurement of the Oil Phase

The viscosity of the oil phase (SORs: 0.5, 0.75, 1.0, 1.25, 1.5, 1.75, 2.0) was determined using a rotational rheometer (Universal Stress Rheometer SR5, Rheometric Scientific). First, the cone and plate fixtures (20 mm) were installed, and the respective temperature set with a water circulator (F25, Julabo GmbH, Seelbach, Germany) and controlled via a temperature controller (Universal Stress Rheometer SR5 Environmental System, Rheometric Scientific). The sample of the respective oil phase was given on the plate, and a dynamic frequency sweep test were performed with the shear rate ranging from 0.01 1/s (initial shear rate) to 10 1/s (end shear rate). The viscosity was determined as slope of the shear stress (output of the rheometer) versus the shear rate. The viscosity of each oil phase was determined in the range of 20–50 with 5 °C increments. For each oil phase, the viscosity at the respective temperature was measured 4 times and the average was calculated. The viscosity of MCT and Tween80 was determined using the same method.

#### 2.2.5 Macrofluidic Emulsification Studies (Burette)

Macrofluidic emulsification studies were conducted by titrating the nonpolar phase in the polar phase to observe the emulsification in a nonmicrofluidic process. The surfactant was either dissolved in the polar phase, i.e., the aqueous phase, or the nonpolar phase, i.e., the oil, prior to the emulsification.

##### Surfactant in the Polar Phase

Tween80 according to the respective SOR (SORs: 0, 0.05, 0.1, 0.25, 0.5, 0.75, 1.0, 1.25, 1.5, 1.75, 2.0) was dissolved in phosphate buffer (5 mm) at 600 rpm for at least 30 min (RCT Basic magnetic stirrer, IKA, Staufen, Germany) in a beaker. The mass of the aqueous phase amounts to 90 g for each experiment. 10 g of MCT was transferred into the burette (*V* = 50 mL, Purex, Corning Inc., Corning, USA), which is positioned in a way that its tip is ≈10 cm above the aqueous phase. MCT is then slowly (droplet by droplet) titrated into the aqueous phase, while the mixture was stirred at 600 rpm. The required titration time was measured. The mixture was then stirred for an additional 10 min at 600 rpm to allow thorough combination. The final emulsions had a mass of 100 g, containing 10 wt% of MCT, and samples were taken. Experiments were conducted a single time as the results align well with literature data.^[^ ^10^ ^]^

##### Surfactant in the Nonpolar Phase

Tween80 according to the respective SOR (SORs: 0, 0.05, 0.1, 0.25, 0.5, 0.75, 1.0, 1.25, 1.5, 1.75, 2.0) was dissolved in 10 g MCT at 600 rpm for at least 30 min (RCT Basic magnetic stirrer, IKA, Staufen, Germany) and then transferred into the burette. The respective mass of phosphate buffer (5 mm) was placed in a 250 mL beaker (final mass of emulsion: 100 g with 10 wt% MCT). The burette is positioned in a way that its tip is ≈10 cm above the aqueous phase. The organic phase was then slowly titrated (droplet by droplet) into the aqueous phase, while the mixture was stirred at 600 rpm. The required titration time was measured. The mixture was then stirred for an additional 10 min at 600 rpm to allow thorough combination, and samples were taken. Experiments were conducted a single time as the results align well with literature data.^[^ ^10^ ^]^ Furthermore, a reproductivity study had been performed changing the height between burette time and the aqueous phase (height: 2.5, 5, 7.5, 10, 12.5, 15, 17.5, 20, 22.5, 25 cm) at SOR of 1.0, which did not show an impact on height on the particle size (see the Supporting Information Experiments), and it was concluded that the experimental setup leads to repeatable results.

#### 2.2.6 Single‐Contact Microfluidic Emulsification Studies

**Figure 3:**
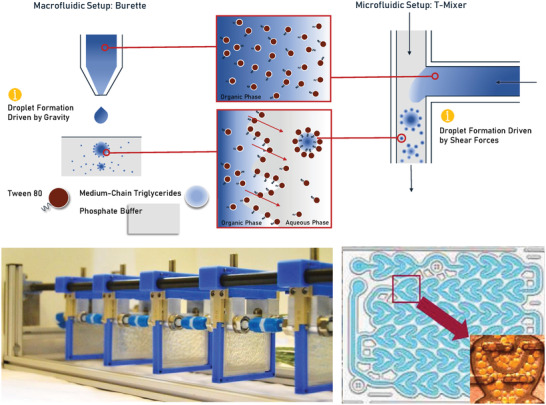
Top: presumed nanodroplet forming fluidics in the burette, left, and T‐microfluidic mixer, right; bottom left: low flow Corning Advanced Reactor system with two pumps; bottom right: heart‐shaped unit cells that are repeated multiple times in the Corning reactor.

Single‐contact microfluidic emulsification studies were conducted by mixing the polar phase and the nonpolar phase using a T‐mixer with an internal diameter of 0.5 mm, see **Figure** **3**. The surfactant was either dissolved in the polar phase, i.e., the aqueous phase, or the nonpolar phase, i.e., the oil, prior to the emulsification.

##### Surfactant in the Polar Phase

Tween80 according to the respective SOR (SORs: 1.0, 2.0) was dissolved in phosphate buffer (5 mm) at 600 rpm for at least 30 min (RCT Basic magnetic stirrer, IKA, Staufen, Germany) in a beaker. The densities of the aqueous phases were determined using the same method as in Section 2.2.3, and is 1.021 g mL^−1^ for SOR = 1.0 and 1.033 g mL^−1^ for SOR = 2.0. Pump 1 (oil phase) and pump 2 (aqueous phase) (both Azura P 4.1S 10 mL, Knauer, Berlin, Germany) were connected via tubing to a T‐mixer (internal diameter = 0.5 mm, Tee LP PEEK 1/4‐28 1/16“ 0.040,” Upchurch Scientific, IDEX Health & Science, Lake Forest, USA) and the outlet of the T‐mixer was connected via tubing to a beaker. Volume flows for pump 1 and pump 2 were set considering the density of the respective aqueous phase and were determined to be 1.067 mL min^−1^ (oil) and 8.933 mL min^−1^ (aqueous phase) for a SOR = 1.0 and 1.077 mL min^−1^ (oil), and 8.923 mL min^−1^ (aqueous phase) for a SOR = 2.0. After 7 min of letting the system reach equilibrium conditions, sample collection started at the outlet: 50 mL of emulsion were collected over the course of 5 min in a beaker, while being stirred at 400 rpm. The emulsion was then stirred for an additional 10 min at 600 rpm to allow thorough combination, and samples were taken. Both experiments were performed a single time.

##### Surfactant in the Nonpolar Phase

Oil phase according to the respective SOR (SORs: 0.05, 0.1, 0.25, 0.3, 0.4, 0.45, 0.5, 0.6, 0.75, 1.0, 1.25, 1.5) was prepared. The mixture was then heated to ≈40 °C while being stirred at 400 rpm to decrease the oil phase's viscosity. Simultaneously, a heat mat (seedling heat mat, 10″ x 20.75,″ T_max_ = 42 °C) was placed around pump 1, and the temperature set to 42 °C. These measures were taken to decrease the viscosity of the oil phase in the inlet and while passing through pump 1. Pump 1 (oil phase) and pump 2 (aqueous phase) (both Azura P 4.1S 10 mL, Knauer, Berlin, Germany) were connected via tubing to a T‐mixer (internal diameter = 0.5 mm, Tee LP PEEK 1/4‐28 1/16“0.040,” Upchurch Scientific, IDEX Health & Science, Lake Forest, USA) and the outlet of the T‐mixer was connected via tubing to a beaker. Once the oil phase and the heat mat reached ≈37–40 °C, volume flows for pump 1 and pump 2 were set considering the density of the respective oil phase, see **Table** **1** Flow rates of microfluidic studies according to the respective SOR of the organic phase. After 7 min of letting the system reach equilibrium conditions, sample collection started at the outlet: 50 mL of emulsion were collected over the course of 5 min in a beaker, while being stirred at 600 rpm. The emulsion was then stirred for an additional 10 min at 600 rpm to allow thorough combination, and samples were taken. Each experiment was performed a single time, as the data aligned with experiment series that were performed under slightly different conditions (see the Supporting Information).

**Table 1:** Flow rates of microfluidic studies according to the respective SOR of the organic phase

SOR [−]	0.05	0.1	0.25	0.3	0.4	0.45	0.5	0.6	0.75	1.0	1.25	1.5
${\dot{V}}_{\mathrm{oil}\;\mathrm{phase}}$ [mL min^−1^]	1.09	1.14	1.27	1.33	1.41	1.46	1.51	1.60	1.74	1.98	2.21	2.46
${\dot{V}}_{\mathrm{aqueous}\;\mathrm{phase}}$[mL min^−1^]	8.91	8.86	8.73	8.67	8.59	8.54	8.49	8.40	8.26	8.02	7.79	7.54
${\dot{V}}_{\mathrm{total}}$ [mL min^−1^]	10	10	10	10	10	10	10	10	10	10	10	10
**Wt**%_**oil**, **outlet**_ [%]	10	10	10	10	10	10	10	10	10	10	10	10

#### 2.2.7 Multicontact Microfluidic Emulsification Studies

Multicontact microfluidic emulsification studies were conducted by mixing the polar phase and the nonpolar phase using a low flow Corning reactor, see Figure 3. The surfactant was either dissolved in the polar phase, i.e., the aqueous phase, or the nonpolar phase, i.e., the oil, prior to the emulsification.

##### Surfactant in the Polar Phase

Tween80 according to the respective SOR (SORs: 1.0, 2.0) was dissolved in phosphate buffer (5 mm) at 600 rpm for at least 30 min (RCT Basic magnetic stirrer, IKA, Staufen, Germany) in a beaker. Pump 1 (oil phase) and pump 2 (aqueous phase) (both Azura P 4.1S 10 mL, Knauer, Berlin, Germany) were connected via tubing to a low flow Corning reactor (Corning Inc., NY). The reduced‐flow reactor consists of 6 modules, i.e., one initial mixing module with 31 hear‐shaped chambers and 5 additional mixing modules with 38 hear‐shaped chambers each, see Figure 3. The number of modules was changed for each experiment, starting with 1 module and increasing the number by one until all 6 modules were installed. The outlet of the reduced‐flow reactor was connected via tubing to a beaker (see Figure 3). Volume flows for pump 1 and pump 2 were set considering the density of the respective aqueous phase and were determined to be 1.067 mL min^−1^ (oil) and 8.933 mL min^−1^ (aqueous phase) for an SOR = 1.0 and 1.077 mL min^−1^ (oil) and 8.923 mL min^−1^ (aqueous phase) for an SOR = 2.0. After 7 min of letting the system reach equilibrium conditions, sample collection started at the outlet: 50 mL of emulsion were collected over the course of 5 min in a beaker. Additional stirring was not necessary. Experiments could only be conducted once as the reactor was blocked by a gel formation over the course of experiments, and although major efforts have been undertaken, the blockage could not be dissolved at the time of submission.

##### Surfactant in the Nonpolar Phase

The multicontact microfluidic emulsification studies were conducted in the same manner as the single‐contact microfluidic emulsification studies, with pump 1 (oil phase) and pump 2 (aqueous phase) connected to the first module of a low flow Corning reactor (Corning Inc., NY). Only oil phases with an SOR of 0.5 and 1.0 were considered (for required flow rates see Table 1). Stirring during sample collection as well as additional mixing after sample collection was not necessary, as the emulsion was already completely combined. Experiments could only be conducted once as the reactor was blocked by a gel formation over the course of experiments, and although major efforts have been undertaken, the blockage could not be dissolved at the time of submission.

#### 2.2.8 Particle Size Analysis

Particle size analysis took place immediately after the experiment was completed to avoid distortion of results due to particle agglomeration or instability mechanisms. The mean particle size (*d*_32_) and the particle size distribution were measured using a static light scattering instrument (Mastersizer 2000 with a Hydro 2000MU Unit, Malvern Instruments Ltd., Malvern, UK). Small amounts of the emulsion samples were added to in the wet sample dispersion unit which had been filled with phosphate buffer (5 mm) to avoid multiscattering effects. A suitable rotational speed of the stirrer in the wet sample dispersion unit had been previously determined to be 750 rpm, as no significant changes of the stirring on the particle size could be observed. The refractive index of MCT is 1.445 according to literature data.^[^ ^10^ ^]^ Each emulsion sample was measured in triplicate, with each measurement generating 3 data sets, leading to a total of at least 9 data sets per sample. The average sample size and particle size distribution were calculated.

## 3 Results and Discussion

### 3.1 Characterization Model System: Ternary Phase Studies

The first target of this study is to identify suitable oil‐water‐surfactant compositions which support the formation of the targeted beverage nanoemulsions. On the one hand, certain compositions can be instable (mixing gaps) and do not show phase dispersion, which needs to be avoided. On the other hand, the formation of gel defines a state of viscosities being too high to be used for further batch or flow processing. Ternary phase diagram studies were conducted to identify areas with targeted degree of phase dispersion. These studies will facilitate to determine suitable range of oil phase concentrations for the emulsification process and will lay the foundation to estimate suitable emulsification process parameters.

The beverage emulsion model system consists of medium‐chain triglycerides as lipid component, Tween 80 as emulsifying agent, and phosphate buffer (5 mm) as aqueous phase. Concentrated beverage emulsions contain >10 wt% oil phase during industrial emulsification, which is then diluted to produce beverage emulsions with ≈0.1 wt% oil phase.^[^ ^25^ ^]^ This requires that the beverages emulsions must be stable over a wide range of concentrations.

Because of the large viscosity difference between the oil and water phase and of the surfactant's ability to dissolve in both phases, it was hypothesized that the order of mixing the components can impact the formation of emulsion. Thus, a design of experiments with three ways of distinct charging‐mixing scenarios were defined, i.e., mixing all three components simultaneously, or premixing the surfactant either with the oil or the water phase before mixing all components together. This defines three experiments, as follows:

① Simultaneous Bulk‐Mix

**Figure 4:**
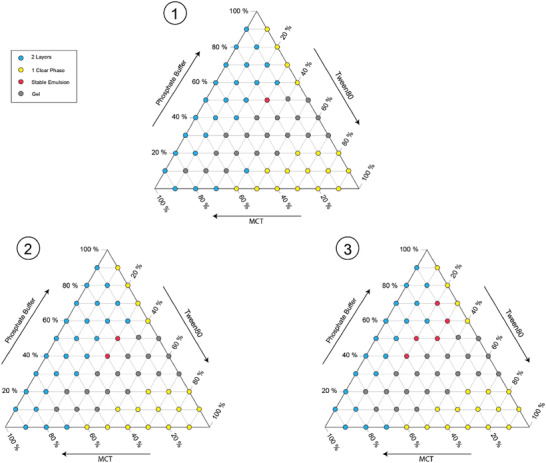
Ternary phase diagrams of a system consisting of Tween 80, Medium‐Chain Triglycerides (MCT), and 5 mmol phosphate buffer (aqueous phase). 1) “Simultaneous bulk‐mix”: all components were mixed at the same time, 2) “Water‐surfactant pre‐mix”: Tween 80 and phosphate buffer were mixed before adding MCT, 3) “Oil‐surfactant pre‐mix”: Tween 80 and MCT were mixed before adding the aqueous phase.

In **Figure** **4**(1), the phase behavior is experimentally determined for an approach in which all components of the model system are simultaneously charged into a vial and mixed via a magnetic stirring bar. This approach represents a simplistic batch process without any premixing. After 7 days, the vials were inspected. When the mixture contained only low concentration of oil and surfactant, opaque mixtures, i.e., emulsions, were formed in the vials at day 0, however, at time of inspection, the mixture was separated in two layers. Only the mixture consisting of 20 wt% MCT, 30 wt% Tween 80, and 50 wt% phosphate buffer did not separate over the course of 7 days, and thus, was considered a stable emulsion. With increasing surfactant concentration, high viscosity of the surfactant hindered thorough mixing in the vial. At surfactant‐to‐buffer ratios exceeding 1:1 in 2‐component mixtures, the formation of a gel‐like material can be observed. Samples were classified as “gel” when the sample's viscosity increased to a point where shaking the vial did not induce a flow or movement of the mixture. In mixtures with high MCT concentrations and low buffer concentrations, the formation of gel persists. At low to mediate MCT concentrations in combination with high surfactant concentrations, the 3‐component mixtures form a single, clear phase of very high viscosity, as the amount of water in the mixture is too low to enable the formation of gel.

②Water‐Surfactant Premix

In Figure 4(2), the experimental results are shown for an approach in which the surfactant was first dissolved in the aqueous phase before adding the oil. This 2‐step emulsification process includes a batch premixing step (aqueous phase and surfactant) followed by a batch mixing step. This was effective to slightly decrease the range of compositions in which a gel forms, as compared to Figure 4(1), particularly at mixtures with comparatively lower surfactant concentrations. In all other compositional areas, either stable or instable emulsions are formed. For example, the mixtures containing 20 wt% MCT, 30 wt% Tween 80, and 50 wt% phosphate buffer, and 30 wt% MCT, 30 wt% Tween 80, and 40 wt% phosphate buffer form a stable emulsion, while the latter being a gel with the charging‐mixing applied in Figure 4b. Thus, a doubling of ternary system compositions that form a stable emulsion has been achieved using the second approach.

③Oil‐Surfactant Premix

In Figure 4(3), a third approach was taken in which the Tween 80 surfactant was first dissolved in the MCT oil before adding the aqueous phase. Equally to Figure 4(2), this approach represents a 2‐step emulsification process with premixing (oil and surfactant) followed by a second mixing of the phases. This forces the surfactant to move during the second mixing from the oil phase to the aqueous phase due to its higher solubility in the latter (hydrophilic affinity). This employs the methodology of spontaneous emulsification. The region in which gel forms is slightly expanded, as compared to Figure 4(2), and resembles the compositional phase areas given in Figure 4(2) except for mixtures containing 10 or 20 wt% aqueous phase. Yet compared to Figure 4(1) and (2), a significant increase in mixture compositions has been achieved forming a stable emulsion, amounting to 4 and 3 data points within the figures, respectively. Using this approach, stable emulsions are obtained at lower surfactant concentrations, i.e., at Tween 80 content of 20 wt% combined with relatively high amount of aqueous phase in the range of 40–90 wt%. This demonstrates that spontaneous emulsification is capable to produce emulsions of target quality, which motivated further investigations presented under Section 3.3.

Proposed composition for flow experiments from the result of the three batch experiments.

Taking the results of the ternary phase studies into account for designing the following experiments, and a potential process concept, it becomes evident that 3‐component compositions within the area of gel formation need to be strictly avoided. The formation of gel poses a serious risk to damage equipment and can cause blockages in tubing and (microfluidic) reactors. Apparently, the order of mixing has little effect on the formation of gel and is dictated by the high concentration of surfactant in combination with low concentrations of the aqueous phase.

Conclusion is to consider a stable 3‐component composition that is not bordering to the gel area, while working in a composition range with low surfactant concentrations and large concentrations of the aqueous phase. This requirement is met by the composition in Figure 4c with 10 wt% MCT, 20 wt% Tween 80 (i.e., surfactant‐to‐oil ratio of 2.0) and 70 wt% phosphate buffer. This composition will be considered as highest surfactant concentration for further investigations. As the surfactant is of no nutritional value, the surfactant concentration will be further decreased for the following experiments, and interesting surfactant‐to‐oil ratios have been defined to be 0.05, 0.1, 0.25, 0.3, 0.4, 0.45, 0.5, 0.6, 0.75, 1.0, 1.25, 1.5, 1.75, and 2.0.

### 3.2 Density and Viscosity of the Oil Phase

Density and viscosity of the oil phase were determined to define the process parameters. This refers to elevated temperature setting to bring these parameters below equipment threshold settings and exclusion of setting those parameters to a range which does not allow proper dosing of small volumes. Further, this defines the flow rates of the continuous‐flow process and tests if mixing of two components can result in the formation of two phases instead of dissolution.

#### 3.2.1 Density of the Oil Phase

In **Table** **2** density of the oil phase in respect to the surfactant‐to‐oil ratio and 5 show the determined viscosities according to the relevant surfactant‐to‐oil ratio. Moreover, the viscosity of MCT was determined to be 0.951 +/− 0.0027 g mL^−1^. The viscosity of Tween 80 was taken from literature data to be 1.064 g mL^−1^.

**Table 2:** Density of the oil phase in respect to the surfactant‐to‐oil ratio

SOR [−]	0.05	0.1	0.25	0.3	0.35	0.4	0.45	0.5
Density [g mL^−1^]	0.958	0.964	0.978	0.977	0.984	0.991	0.995	0.993
Standard deviation [g mL^−1^]	0.0022	0.0022	0.002	0.0019	0.0027	0.0017	0.0026	0.0023

SOR [−]	0.6	0.75	1.0	1.25	1.5	1.75	2.0
Density [g mL^−1^]	1.002	1.005	1.015	1.023	1.022	1.025	1.029
Standard deviation [g mL^−1^]	0.0028	0.0019	0.0024	0.003	0.002	0.0045	0.004

**Figure 5:**
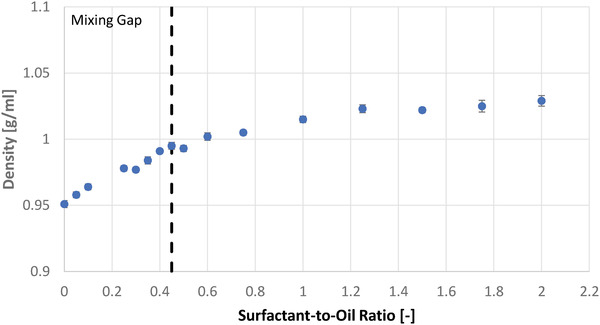
Density of the oil phase in respect to the surfactant‐to‐oil ratio.

The density of the oil phase increases with increasing Tween 80 concentration. The viscosity increased with increasing Tween 80 concentration. Due to the higher viscosity, it was difficult to accurately dose the oil phase from the syringe into the measurement container, which might account for the higher standard deviation at SOR = 1.75 and SOR = 2.0. It was observed during the measurements that the surfactant did not dissolve completely into the oil below SOR of 0.5 but forming an opaque mixture instead which separated when not stirred. Thus, this area is considered a mixing gap at ambient temperature, and oil phases with SOR ≤ 0.45 were stirred during sample removal. Recognizing the mixing gap in **Figure** **5**, the densities increase at a higher rate within the mixing gap compared to outside the mixing gap.

The determined densities were used to define flow rates for the following microfluidic experiments. In reference to the macrofluidic emulsification studies, flow rates were specified to obtain emulsions containing 10 wt% MCT in the outlet.

#### 3.2.2 Viscosity of the Oil Phase

Oil phases with increasing Tween 80 content have higher viscosities compared to the bulk oil. High viscosities can lead to difficulties during the emulsification, as certain equipment is designed to withstand only a certain range of viscosities. In our case, the pumps 1 and 2 used for single‐contact and multicontact microfluidic emulsification studies are built to handle liquids of up to 100 mPa s at up to 40 °C. The viscosities of each MCT and Tween 80 at temperatures between 20 and 50 °C were determined. MCT shows viscosities well below the equipment‐imposed viscosity limit throughout all temperatures, specifically the viscosity decreases from 31.7 mPa s at 20 °C to 12.6 mPa s at 50 °C. Yet, Tween 80 has a significant higher viscosity with 727.9 mPa s at 20 °C. Although there is a strong decline in viscosity with increasing temperature, the surfactant's viscosity is still at 183.6 mPa s at 50 °C, and therefore well above the equipment‐imposed viscosity limit.

**Figure 6:**
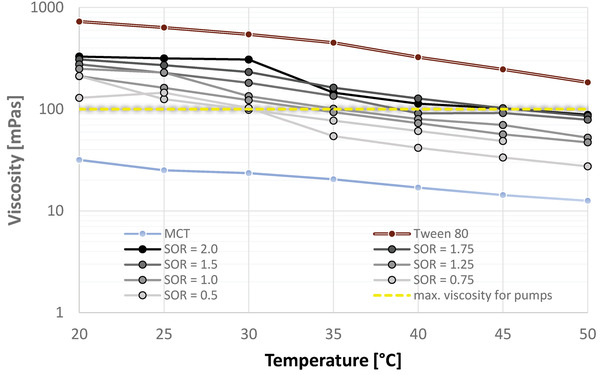
Viscosity of oil phases with SOR = 0.5–2.0, MCT, and Tween 80 in respect to temperature.

In **Figure** **6**, the viscosities of oil‐surfactant mixtures with SOR between 0.5 and 2.0 were determined as a function of temperature. Oil‐surfactant mixtures with SORs below 0.5, i.e., within the mixing gap, were not considered, because a two‐phase system complicates the viscosity measurements, and their viscosities were qualitatively conceived to be close to the viscosity of MCT due to their relatively low surfactant content. The viscosity of the investigated mixtures increases with increasing surfactant content, and range from 129.4 mPa s (SOR = 0.5) to 329.2 mPa s (SOR = 2.0) at 20 °C. The viscosity of all mixtures decreases with increasing temperature at a similar decline. Some SOR data points are above or below the viscosities of mixtures with neighboring SORs, yet this might be referred to equipment limitation with a standard deviation ranging from 0.6% to 13% for different data points.

The decrease in viscosity of a given oil phase with an SOR between 0.5 and 2.0 with temperature was found best to be described with a logarithmic relation, as it resulted in the highest correlation coefficient for each SOR

(2) $$\eta =-a*\ln\left(T\right)+b$$

With *η* being the dynamic viscosity [mPa s], T being the temperature [°C), and a and b being constant values respective to the SOR. It was assumed that the constant values *a* and *b* are dependent on the surfactant to oil ratio, and the relationship of each a and b to the SOR was estimated using linear correlation

(3) a=99.87∗SOR+98.26

(4) b=444.96∗SOR+359.77

Leading to the following correlation

(5) $$\begin{array}{c} \eta =-\left(99.87*SOR+98.26\right)*\ln\left(T\right)+\left(444.96*SOR+359.77\right) \end{array}$$

Equation (5) gives the viscosity of a given oil phase with an SOR between 0.5 and 2.0 within the temperature window of 20–50 °C with an approximate deviation of up to ± 20 mPa s.

At 50 °C, all mixtures have viscosities below 100 mPa s, i.e., 27.4 mPa s (SOR = 0.5) to 88.2 mPa s (SOR = 2.0), yet this temperature is above the equipment‐imposed temperature limit. Thus, for the following experiments, the focus is laid at the temperature of 40 °C, lowering the viscosity of most oil‐surfactant mixtures below 100 mPa s, i.e., 41.73 mPa s (SOR = 0.5), 61.05 mPa s (SOR = 0.75), 71.82 mPa s (SOR = 1.0), 80.13 mPa s (SOR = 1.25), and 91.18 mPa s (SOR = 1.5). Therefore, the highest SOR which can be considered in microfluidic studies is 1.5, and mixtures with SOR of 1.75 and 2.0 need to be excluded to prevent damage to the equipment.

#### 3.2.3 Recommendations for Microfluidic Experiment

To conduct the microfluidic experiments, the oil‐surfactant mixture is heated at the pump inlet, and the pump itself is wrapped with a heating mat. It is assumed that the heating of the oil phase has a negligible influence on the emulsification process, as the channels of the pump's outlet are not heated. In view of the channel length of ≈20 cm for the T‐Mixer and 40 cm for the reduced‐flow reactor it can be assumed that the oil phase has potentially cooled down to ambient temperature once it reaches the microfluidic reactor.

### 3.3 Evaluation of Spontaneous Emulsification and Microfluidic Mixing

A batch approach provides small specific oil/water‐interfacial area that could hinder the spontaneous emulsification. Stirring might not overcome the large viscosity of the oil phase added in one step, which would prevent the formation of small droplets. Continuous‐flow emulsification processes allow to add the oil phase in steps, i.e., by small microfluidic volumes, to the aqueous phase, which creates large specific interfacial areas. Assuming that interfacial area is the limiting step in spontaneous emulsification, this might provide potential to decrease the required surfactant concentration to form a stable emulsion.

For the following experiments the oil amount is defined to 10 wt% for the final emulsion, while the respective surfactant concentration is varied to reach surfactant‐to‐oil ratios between 0.05 and 2.0. The design of the experiments aims to identify the potential of 1) spontaneous emulsification compared to the conventional emulsification, and 2) microfluidic mixing devices compared to macrofluidic devices.

#### 3.3.1 Macrofluidic Experiments

**Figure 7:**
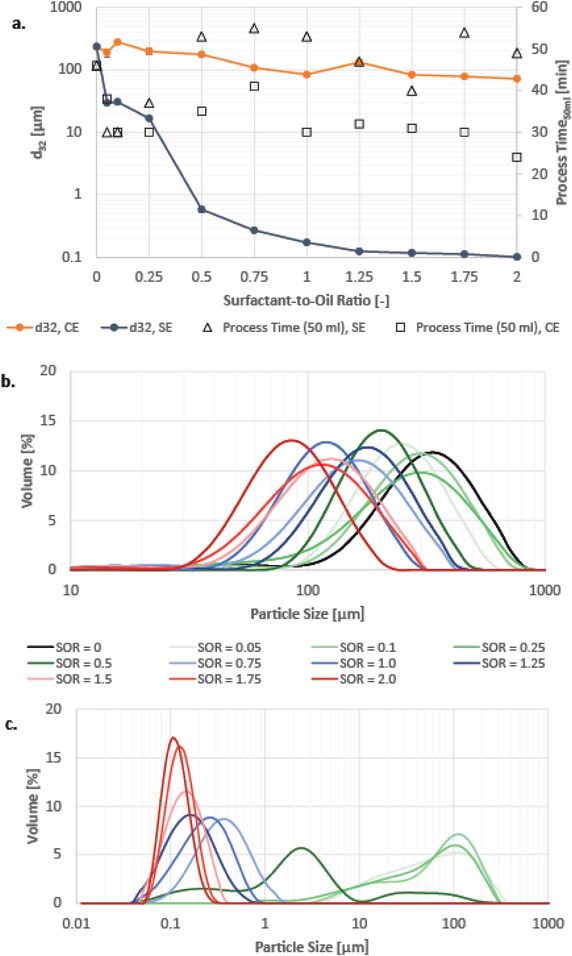
a) Sauter Diameter in respect to surfactant‐to‐oil ratio (CE: conventional emulsification, SE: spontaneous emulsification). b) Particle size distribution of macrofluidic‐made emulsions using conventional emulsification. c) Particle size distribution of macrofluidic‐made emulsions using spontaneous emulsification.

**Figure** **7**a shows the Sauter mean diameter *d*_32_ of the model system emulsions formed through either conventional emulsification or spontaneous emulsification over varying SORs. Without surfactant, a droplet diameter of 235 µm is derived by titrating the oil in the aqueous phase and stirring the mixture at 600 rpm, and oil and the aqueous phase separated upon stopping the stirring. Regarding the conventional emulsification, emulsions with droplet diameters between 190 and 280 µm are obtained with minimal surfactant concentration, i.e., SOR ranges from 0.05 to 0.25. With increasing SOR the Sauter mean diameter decreases to a value of 72 µm at SOR = 2.0. During experimentation, it was observed that emulsions with SOR of up to 1.25 were unstable, i.e., a part of the droplets quickly rose to the surface when stirring stopped. Thus, these emulsions were stirred during sample removal for particle size measurements, and these samples quickly transferred to the measurement unit. Yet overall, the particle sizes of emulsions obtained through the conventional emulsification are in the range of micrometers, and therefore do not suit the targeted nanoparticle range.

The Sauter mean diameter of all emulsions of spontaneous emulsification is below that of emulsions made by conventional emulsification. Even at low surfactant concentration (SOR = 0.05–0.25) droplets had smaller diameter (*d*_32_ = 17–31 µm) than the smallest diameter of conventional‐made emulsions (*d*_32_ = 72 µm). With increasing surfactant concentration, the Sauter mean diameter decreases strongly until it reaches a value of 172 nm at SOR = 1.0. Above SOR of 1.0 the decline is slower, and from an SOR of 1.25, there is only a small decrease in particle size with Sauter mean diameters decreasing from 117 nm (SOR = 1.5) to 101 nm (SOR = 2.0). All spontaneous‐made emulsions were stable without showing creaming. Thus, according to the common definition of *d*_32_ = 200 nm for nanoemulsions, the targeted range was achieved at SOR of 1.0. At SOR = 1.0 and SOR = 2.0, the Sauter mean diameter is decreased by 99.8% and 99.9%, respectively, for spontaneous emulsification over conventional emulsification. The results regarding the particle size of conventional and spontaneous emulsification align with the findings of Komaiko et al. on a smaller scale (*V*_final emulsion_ = 50 mL) using an automated burette.^[^ ^10^ ^]^

Regarding the particle size distribution of macrofluidic‐made emulsions using either the conventional emulsification (see Figure 7b) or the spontaneous emulsification (see Figure 7c), both times the particle size distribution shifts to smaller particle size values with increasing surfactant concentration, in accordance with their Sauter mean diameters. While the particle size distributions of emulsions obtained with conventional emulsification resembles a normal distribution at all surfactant concentrations, the particle size distributions of emulsions obtained with spontaneous emulsification do only resemble a normal distribution at relatively high surfactant concentration, i.e., above an SOR of 0.75. Below this value, the particle size distributions are asymmetrical and cover a large range of particle sizes.

#### 3.3.2 Single‐Contact Microfluidic Mixer

The process time (see Figure 7a) is defined as the time to form 100 mL of emulsion including additional stirring. The process time of the spontaneous emulsification is in most cases well above the process time of the conventional emulsification, which can be traced back to the different volumes of oil phase that needed to be titrated in the aqueous phase (10 g per conventional emulsions vs 10.1–20.0 g per spontaneous emulsion). The dripping speed was deliberately set slower to allow sufficient mixing, as initial jellification could be observed when oil phase droplets came in contact with the aqueous phase. It was difficult to set accurate dripping speeds with the manual burette, which explains the strong variation of process times, and might also depend on the individual person performing the experiment.

**Figure 8:**
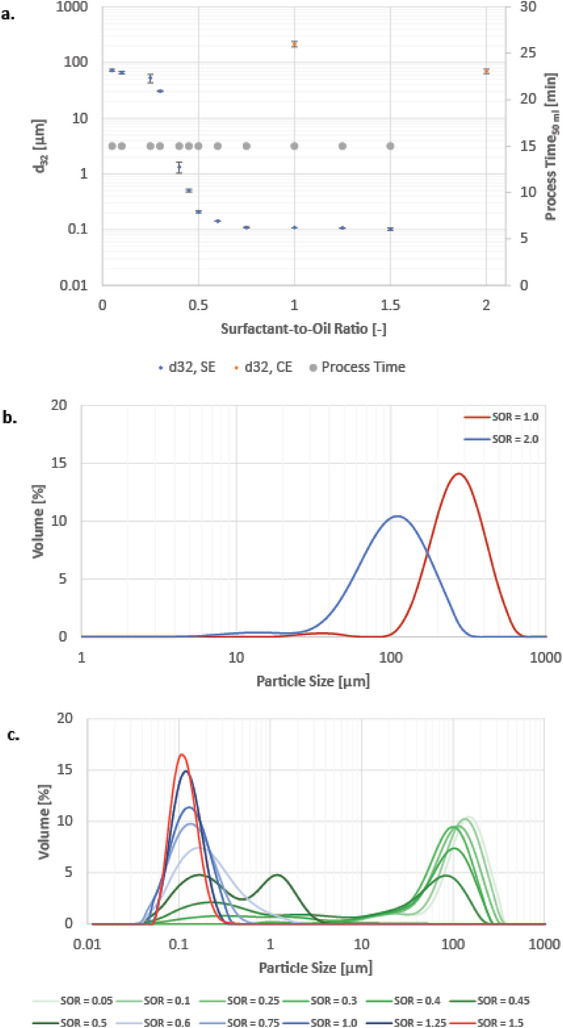
a) Sauter mean diameter in respect to the surfactant to oil ratio (CE conventional emulsification, SE: spontaneous emulsification). b) Particle size distribution of single‐contact microfluidic‐made emulsions using conventional emulsification. c) Particle size distribution of single‐contact microfluidic‐made emulsions using spontaneous emulsification.

**Figure** **8**a shows the Sauter mean diameter *d*_32_ of the model system emulsions formed either through conventional emulsification or spontaneous emulsification over varying SORs. The conventional emulsification has been investigated at a SOR of 1.0 and 2.0 to confirm previous observations. The particle size is within the range of tens to hundreds of micrometers, and thus in the same range as the conventionally made, macrofluidic emulsions. At a SOR of 2.0, approximately the same particle size was obtained at both experimental setups (*d*_32_ = ≈70 µm), while at an SOR of 1.0 the Sauter mean diameter of the single‐contact microfluidic mixer is 214 µm, and thus about 2.5 times as large as the Sauter mean diameter of conventional emulsions obtained with the macrofluidic experimental setup.

Regarding the spontaneous emulsification, the particle size at very low surfactant concentrations (SOR = 0.05–0.1) equals approximately the particle size of the conventional emulsification at SOR of 2.0, i.e., ≈70 µm. With increasing surfactant concentrations, *d*_32_ decreases relatively slowly from to 31 µm at low surfactant concentrations (SOR = 0.05–0.30). Between SOR = 0.30–0.50 the particle size decreases strongly, and reaches 209 nm at SOR = 0.50. Between SOR = 0.50–0.75, the particle size decreases further, yet at a lower rate, and reaches 109 nm at SOR = 0.75. Above SOR of 0.75, the Sauter mean diameter does not significantly change. Surfactant concentrations above SOR = 1.50 could not be studied, as previous measurements had shown that the viscosities of the according oil phases are above the working range of the pumps used (see Section 2.2.4). The microfluidic process exhibits a strong influence of the surfactant concentration on the Sauter mean diameter as does the batch process. Compared to the macrofluidic process, the particle diameter of microfluidic made emulsions is approximately twice as large at low surfactant concentration. The decrease in particle size is stronger in microfluidic made emulsions, leading to a considerably smaller particle size at mediate surfactant concentrations. The targeted particle diameter for nanoemulsions (≈200 nm) is reached at SOR = 0.50 compared to SOR = 1.00 at macrofluidic made emulsions, thus decreasing the required surfactant use by 50%.

Regarding the particle size distribution of single‐contact microfluidic‐made emulsions using either the conventional emulsification (see Figure 8b) or the spontaneous emulsification (see Figure 8c), in conjunction with observations under Section 3.3.1, the particle size distribution shifts to smaller particle size values with increasing surfactant concentrations, in accordance with their Sauter mean diameters. For the conventional emulsification, the particle size distribution becomes wider with increasing surfactant concentration. In general, narrow particle size distributions are favorable as they tend to lead to emulsions with increased stability. Regarding the spontaneous emulsification, a shift of the particle size distribution to smaller particle sizes can also be observed, yet, instead of a continuous shift there is a leap: While an SOR of up to 0.4 leads to single particle size distribution (PSD) peaks at around 100–160 µm, at an SOR of 0.45 and 0.5 bimodal distribution that spans over a wide range of particle sizes. Particle size distribution upward of a SOR of 0.6 center approximately at around 0.1–0.16 µm and become narrower with increasing surfactant concentration.

In summary, instead of a monomodal PSD continuously shifting toward smaller particle sizes with increasing surfactant concentration as observed with the conventional emulsification, there is a transition area at moderate surfactant concentrations when applying the spontaneous emulsification. In this transition area with moderate surfactant concentrations, a bi‐modal PSD can be observed, while at high and low surfactant concentrations, a monomodal PSD prevails. Similar observations can be made in Section 3.3.1 at moderate surfactant concentrations, thus indicating this observation might be connected to the spontaneous emulsification rather than the microfluidic mixing. Potentially, different underlying emulsification mechanisms prevail at low or high surfactant concentrations, respectively, whereas at moderate surfactant concentrations both mechanisms take place, leading to a bi‐modal PSD. Yet, the respective mechanisms are unknown, and indicate that further investigation is required.

Based on these observations, it might be favorable to increase the required surfactant concentrations to an SOR of 0.6 in order to obtain a narrower, monomodal particle size distribution, and consequently higher stability. Still, compared to macrofluidic made emulsions, the required surfactant use is decreased by 40%.

The process time to form 50 mL of emulsion by a microfluidic T‐mixer amounts to 15 min for both the conventional and spontaneous emulsification (see Figure 8a), not including the start‐up time required to reach equilibrium conditions. In contrast to the macrofluidic experiments, the process time was not dependent on the changing ratio of oil phase to aqueous phase, which was catered for by adjusting the volume flow, and by the pumps to achieve a stable volume flow of fluids with different viscosities. Since the overall volume flow was set to 10 mL min^−1^, it took 5 min for every experiment to obtain 50 mL of mixture in the mixture.

Initial experiments showed that the oil phase and the aqueous phase were not completely combined when leaving the single‐contact microfluidic mixer, leading an emulsion with either gel‐like residue at the bottom of the outlet beaker or an oil film on top of the emulsion in case of spontaneous emulsification or conventional emulsification, respectively. Thus, each emulsion was stirred for an additional 10 min after microfluidic mixing, which led in all cases to a completely combined emulsion.

Although the overall process time is significantly decreased by single‐contact microfluidic mixing compared to the macrofluidic mixing (by ≈50%), the process time is not yet on a practical, competitive level for nanoemulsion production, especially for a beverage emulsion. While the emulsion formation is fast and profits further from rapid dilution by a factor of 100 to reach oil contents of beverage nanoemulsions (≈0.1 wt%), the period of additional mixing of 10 min prevails and does not align with the convenience idea of an on‐demand beverage dispenser. Moreover, additional stirring would require for the process setup to be expanded with an additional stirring tank, requiring more equipment for the overall process.

#### 3.3.3 Multicontact Microfluidic Mixer

The multicontact microfluidic mixer operates in a re‐entrance flow regime, meaning the multiphase mixture is never in equilibrium, but constantly perturbed, providing large surface renewal and large convectional transport to the surface at high specific interfacial area and away from it. The surface renewal should favor the transport of a large amount of surfactant through the interface, and convection within the phases ensures that the mass transport to and from the interface can cope with its speed of renewal.

**Figure 9:**
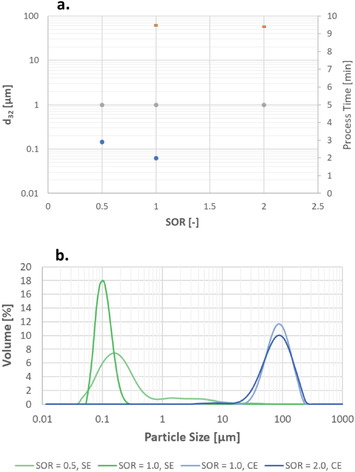
a) Sauter diameter and process time in respect to surfactant‐to‐oil ratio. b) Particle size distribution of multicontact microfluidic‐made emulsions using both spontaneous emulsification (SOR = 0.5, 1.0) and conventional emulsification (SOR = 1.0, 2.0).

**Figure** **9**a shows the Sauter mean diameter and the process time of the spontaneous emulsification and the conventional emulsification using a low flow Corning reactor (1 module) at surfactant‐to‐oil ratios of 0.5 and 1.0 as well as 1.0 and 2.0, respectively. Regarding the conventional emulsification, the particle sizes are within the range of 57–61 µm, and no significant influence of the surfactant concentration on the particle size and the particle size distribution (see Figure 9b) is visible. Compared to macrofluidic and single‐contact microfluidic droplet generation, multicontact microfluidic droplet generation leads to the overall smallest particle sizes for the conventional emulsification, regardless of surfactant concentration. Later in this section, the influence of the number of modules in the low flow Corning reactor on emulsion characteristics will be discussed.

Regarding the spontaneous emulsification, at SOR of 0.5 a Sauter mean diameter of 144 nm was obtained. Compared to the macrofluidic and single‐contact microfluidic setup, this represents a decrease in droplet size of 75% and 31%, respectively. At SOR of 1.0 a Sauter mean diameter of 94 nm was obtained. Because the diameter is at the lower measurement limit of the static light scattering instrument, this measurement was confirmed with a dynamic light scattering device (Zetasizer, Malvern Instruments Ltd., Malvern, UK), and a Z‐average of 62 nm was obtained. This compares to the macrofluidic and single‐contact microfluidic setup as a decrease in droplet size of 64% and 42%, respectively. Therefore, the low flow Corning reactor led to the smallest particle size for the spontaneous emulsification in respect to surfactant concentration, and to the overall smallest particle size obtained throughout the emulsification experiments. The results suggest that the surfactant concentration could be further decreased to reach the targeted droplet size of *d*_32_ = 200 nm compared to using the single‐contact microfluidic mixer for emulsification.

Regarding the particle size distribution using spontaneous emulsification, the PSD becomes narrower with increasing surfactant concentration. Compared to the single‐contact microfluidic mixer, there is no bimodal particle size distribution at an SOR of 0.5, but a quasimonomodal curve, which extents toward higher particle sizes. This finding could indicate that an emulsion with a SOR of 0.5 might be more stable when produced from a multicontact microfluidic reactor than from a single‐contact microfluidic reactor, as monomodal PSDs are linked with a higher emulsion stability. Investigations on the emulsion stability in respect to particle size and PSD are favorable.

Originally, further experiments were planned that consider more SOR values, particularly for the spontaneous emulsification. It was anticipated to identify the impact of low, moderate, and high surfactant concentration on the emulsion characteristics, specifically to identify the surfactant concentration required to obtain a Sauter diameter of 200 nm (targeted droplet size). Moreover, considering the bimodal PSD at moderate surfactant concentrations with the single‐contact microfluidic mixer, it would be worthwhile to investigate the potential occurrence of a bimodal PSD at certain surfactant concentrations with the multicontact microfluidic mixer. Yet unfortunately, anticipated experiments were prevented from a blockage in the low flow Corning reactor. The blockage was caused by jellification of the oil phase, which was considered in 3.1 as major risk for the process design, and took place during cleaning of the mixer. It is assumed that during purging of the reactor, unfavorable ratios between oil phase and aqueous phase formed, leading to jellification of the mixture, and thus a complete blockage. Although major efforts have been made to dissolve the gel, only parts of the module could be cleaned, and the blockage remains at the time of manuscript submission, preventing to conduct further experiments. The experiments are anticipated once the blockage is dissolved, and the equipment is back in full working conditions. However, this represents a major drawback to this mixing approach, as blockages disrupt the production process, and represent a hazard, as the sudden pressure increase might destroy the equipment. Certainly, cautious cleaning procedures needs to be established to avoid risky oil phase concentrations at all times.

Regarding the process time, the process time was not dependent on the oil phase to aqueous phase ratio, therefore corresponding to the single‐contact microfluidic experiments. The volume flow was adjusted accordingly, while the pumps ensured a stable flow within a certain range of viscosities. The overall volume flow was set to 10 mL min^−1^ (maximum volume flow for low flow Corning reactor), resulting in a process time of 5 min for a volume of 50 mL emulsion (see Figure 9a) both for conventional and spontaneous emulsification, not including the start‐up time required to reach equilibrium conditions. Contrary to the single‐contact microfluidic mixer, no additional stirring was required, as the emulsion was already completely combined at the outlet. Thus, using the multicontact microfluidic device led to the overall shortest process time, equal to a reduction in process time of 83% or 67% compared to the macrofluidic and the single‐contact microfluidic mixing setup, respectively. Considering the factor of dilution, the process time for a single serving of a beverage with a volume of 250–300 mL (oil content of 0.1 wt%) requires 2.5–3 mL of concentrated emulsion (oil content of 10 wt%), which would take 15–18 s using the low flow Corning reactor. Thus, the process time is now within a competitive scale for the production of beverage emulsions in the case of on‐demand beverage dispensers.

**Figure 10:**
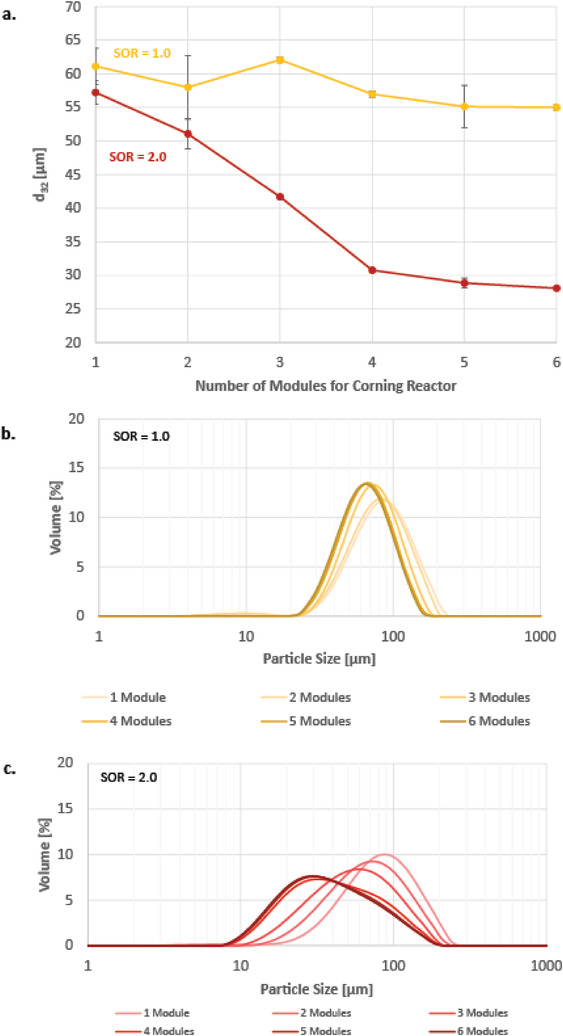
a) Sauter diameter in respect to the number of Corning microfluidic modules (low flow Corning reactor). b) particle size distribution at SOR = 1.0 the number of modules installed in the reduced‐flow reactor. c) particle size distribution at SOR = 2.0 in respect to the number of modules installed in the reduced‐flow reactor.

**Figure** **10** shows the particle size of the conventional emulsification in respect to the number of modules installed in the low flow Corning reactor. The first module is designed for the initial encounter of both phases including subsequent convection mixing in the characteristic heart chambers, while any additional module installed prolongs the convection mixing by providing additional 38 heart chambers per module. While at SOR of 1.0 there is no significant influence of the number of mixing modules on the droplet size, the droplet size changes significantly with the number of modules at SOR of 2.0, see Figure 10. The Sauter mean diameter drops from 57 µm at one module by ≈50% to 31 µm at 4 modules. Increasing the number of modules further to 5 or 6 units only decreases the Sauter mean diameter by 2 or 3 µm, respectively, and is thus not considered as a significant decrease in particle size. With increasing number of modules installed in the reduced‐flow reactor, the particle size distribution becomes slightly narrower at a SOR of 1.0 (see Figure 10b). At an SOR of 2.0, the particle size distribution shifts towards smaller particle sizes, yet becomes wider with increasing number of modules (see Figure 10b,c). Therefore, providing additional convection mixing can decrease the particle size, but only within the narrow range of targeted surfactant concentration. Although the low flow Corning reactor generated the smallest particle sizes for the conventional emulsification, all generated emulsions are well above the targeted range of nanodroplets. Again, it is anticipated to repeat these experiments using the spontaneous emulsification to investigate the impact of the number of modules on the emulsion's characteristics.

## 4 Conclusions 

This study demonstrated that continuous‐flow is capable and advantageous for forming nanoemulsification via spontaneous self‐assembly, and deciphers the role specific microfluidics have. Multicontact microfluidics show strong advantages above other systems when combined with spontaneous emulsification, with droplet sizes of 62 nm at SOR = 1.0 and a decrease of 90% in process time. Microfluidic spontaneous emulsification meets, therefore, the specific demands of the application, which are to provide fortified beverages to astronauts in space exploration. Multicontact microfluidics also showed drawbacks by jellification and related cleaning issues that were not found for single‐contact microfluidics.

The better performance of continuous‐flow techniques is explained by the smaller and more uniform size of the initial micrometer‐sized droplets, i.e., larger interfacial area, as compared to pipetting into a tank. Individual continuous‐flow techniques differ in their quality of interface renewal (droplet‐to‐bulk) and convection (vortex‐mixing) within droplets, which is why the multicontact microfluidic mode performed better. Continuous‐flow could also ensure absence of undesired phases, including gels.

The formation of stable nanoemulsions was achieved at a level of concentrations much higher as compared to application (human intake). Ternary phase diagram analysis of the three components (oil, water, surfactant) allowed to decide for the right mixing ratio and sequence of mixing steps for the nanoemulsions. This prevented to form instable or undefined (inhomogeneous) nanoemulsions. The microfluidically made nanoemulsions were amenable to dilution to the final beverage emulsions, without loss of quality at a dilution factor of up to 100. This allowed to speed up the process time needed for a standard beverage volume, since the dilution can be accomplished without notable time delay. This is an additional advantage for an in‐space manufacturing and application for human space exploration. An expresso takes 25–30 s to be made and people are accustomed with this wait time, our system might be even faster which exceeds expectations. The high oil loads may even provide opportunity to supply the nanoemulsions as payloads to a spacecraft or space habitat, and then diluted on the spot.

The surfactant content was reduced to meet healthcare compliance, and to account for a higher use of fortified beverages by astronauts as compared to an Earth consumer. Microfluidic emulsification demonstrated to produce suited nanoemulsions at lower surfactant concentrations than macrofluidic emulsification. Microfluidic emulsification can be done in single‐contact mode. Constant process conditions are established after initial setting. Multicontact microfluidic processing demonstrated to be more efficient to deliver targeted nanoemulsification quality, yet also showed drawbacks by jellification and related cleaning issues.

Key conclusion is that the combination of spontaneous emulsification and microfluidics leads to a more efficient process compared to each technology on their own, in terms of surfactant efficiency, overall process time, and droplet size.

## 5 Outlook

Given the positive results regarding the particle size, but the unexpected observations in terms of particle size distribution when applying spontaneous emulsification, we plan a future investigation into the mechanisms of the spontaneous emulsification at varying surfactant‐to‐oil‐ratios. Particularly, it will be interesting to investigate whether the transition area with bi‐modal PSDs can also be observed using a multicontact microfluidic mixer. We intent to conclude the experiments using a multicontact microfluidic mixer over the whole range of SORs once, we overcome the equipment issues caused by blockage through oil phase jellification. Ultimately, we intent to contribute to the understanding of the mechanism of the spontaneous emulsification in general, and to the understanding of the spontaneous emulsification mechanism in microfluidics in particular.

Furthermore, experiments with multicontact microfluidic mixers of similar mixing performance with higher maximum volume flow are anticipated, e.g., the G1 Corning Reactor of a similar design and maximum volume flows of 300 mL min^−1^, which could decrease the process time further.^[^ ^26^ ^]^

In addition, the use of applying a constant force for improving mass and interfacial transport over the whole reactor length is considered. The coiled flow inverter (CFI) comprises a coiled capillary which after four turnings inverted for coiling direction (clockwise, counterclockwise).^[^ ^27^, ^28^ ^]^ Dean forces are created which promote mass recirculation and interfacial transport in single and multiphase microfluidic flows. We have used in the past the CFI for minerals extraction, for Earth applications^[^ ^29^, ^30^ ^]^ and for space (Asteroid minerals extraction).^[^ ^31^ ^]^

The incorporation of nutrients and medicines in the nanodroplets has to be demonstrated, and the stability of the nanoemulsions. Likely, some adjustments in the microfluidic manufacturing has to be made to ensure beverage quality. We also like to add flavors to the nanoemulsions to improve the sensory quality to the beverages.

Finally, we will automate the microfluidic system, which means to add process monitoring sensors and analytics (e.g., for temperature, pressure, particle size control) and valves for choosing and dosing various nutrients and medicines out of a chemical stock library. This will allow to customize the contents of a fortified beverage, in the sense of a “designer beverage.” Pumps, valves, and monitoring units will be controlled by a computer, which will also allow a remote operation of a microfluidic processing unit for beverages.

## Conflict of Interest

The authors declare no conflict of interest.

## Supporting information

Supporting Information

## Data Availability

The data that support the findings of this study are available in the supplementary material of this article.
