# Supplementary material for: Microfluidic Spontaneous Emulsification for Generation of O/W Nanoemulsions—Opportunity for In‐Space Manufacturing
Source: Adv Healthc Mater. 2023 May 4;12(23):2203363. doi: 10.1002/adhm.202203363 (PMC11468665; doi:10.1002/adhm.202203363)
Supplement: Supplementary file 1 — Supporting Information [file ADHM-12-2203363-s001.pdf]

ADVANCED HEALTHCARE MATERIALS

Supporting Information

for *Adv. Healthcare Mater.*, DOI 10.1002/adhm.202203363

Microfluidic Spontaneous Emulsification for Generation of O/W Nanoemulsions—Opportunity for In-Space Manufacturing

*Svenja Schmidt, Anh The Nguyen, Huy Quang Vu, Nam Nghiep Tran, Maria Sareela, Ian Fisk and Volker Hessel**

Supplemental Material: Microfluidic Spontaneous Emulsification for Generation of O/W Nanoemulsions - Opportunity for In-Space Manufacturing

1. Composition of tertiary phase diagram samples

Table 1: Composition of tertiary phase diagram samples consisting of 3 components (phosphate buffer 5 mM, MCT, Tween 80).

# Vial	wt%$_{Phosphate\ Buffer}$	wt%$_{MCT}$	wt%$_{Tween\ 80}$	# Vial	wt%$_{Phosphate\ Buffer}$	wt%$_{MCT}$	wt%$_{Tween\ 80}$
**1**	1	0	0	**34**	0.4	0.3	0.3
**2**	0.9	0.1	0	**35**	0.3	0.4	0.3
**3**	0.8	0.2	0	**36**	0.2	0.5	0.3
**4**	0.7	0.3	0	**37**	0.1	0.6	0.3
**5**	0.6	0.4	0	**38**	0	0.7	0.3
**6**	0.5	0.5	0	**39**	0.6	0	0.4
**7**	0.4	0.6	0	**40**	0.5	0.1	0.4
**8**	0.3	0.7	0	**41**	0.4	0.2	0.4
**9**	0.2	0.8	0	**42**	0.3	0.3	0.4
**10**	0.1	0.9	0	**43**	0.2	0.4	0.4
**11**	0	1	0	**44**	0.1	0.5	0.4
**12**	0.9	0	0.1	**45**	0	0.6	0.4
**13**	0.8	0.1	0.1	**46**	0.5	0	0.5
**14**	0.7	0.2	0.1	**47**	0.4	0.1	0.5
**15**	0.6	0.3	0.1	**48**	0.3	0.2	0.5
**16**	0.5	0.4	0.1	**49**	0.2	0.3	0.5
**17**	0.4	0.5	0.1	**50**	0.1	0.4	0.5
**18**	0.3	0.6	0.1	**51**	0	0.5	0.5
**19**	0.2	0.7	0.1	**52**	0.4	0	0.6
**20**	0.1	0.8	0.1	**53**	0.3	0.1	0.6
**21**	0	0.9	0.1	**54**	0.2	0.2	0.6
**22**	0.8	0	0.2	**55**	0.1	0.3	0.6
**23**	0.7	0.1	0.2	**56**	0	0.4	0.6
**24**	0.6	0.2	0.2	**57**	0.3	0	0.7
**25**	0.5	0.3	0.2	**58**	0.2	0.1	0.7

**26**	0.4	0.4	0.2	**59**	0.1	0.2	0.7
**27**	0.3	0.5	0.2	**60**	0	0.3	0.7
**28**	0.2	0.6	0.2	**61**	0.2	0	0.8
**29**	0.1	0.7	0.2	**62**	0.1	0.1	0.8
**30**	0	0.8	0.2	**63**	0	0.2	0.8
**31**	0.7	0	0.3	**64**	0.1	0	0.9
**32**	0.6	0.1	0.3	**65**	0	0.1	0.9
**33**	0.5	0.2	0.3	**66**	0	0	1

2. Reproductivity study of the burette experimental setup

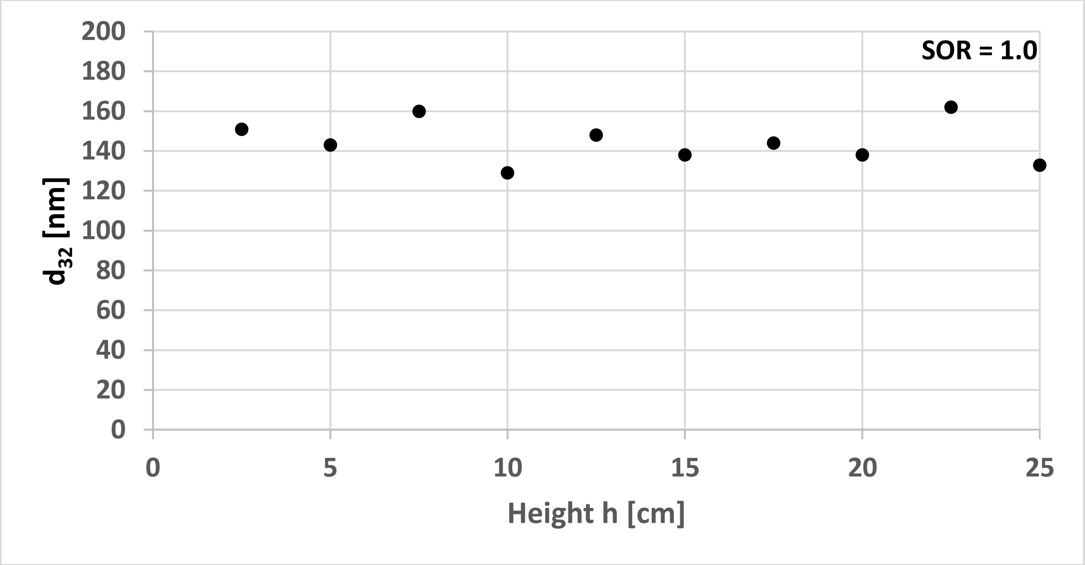

Figure 1: Influence of height h between burette tip and the surface of the aqueous phase on the particle mean diameter d_{32}. Surfactant Tween80 was initially dissolved in the non-polar phase at a surfactant-to-oil ratio of 1.0. The experiments were conducted so that the final emulsion had a weight of 100 g and contained 10 g of medium-chain triglycerides.

3. Additional Result from single-contact microfluidic mixer under varying conditions

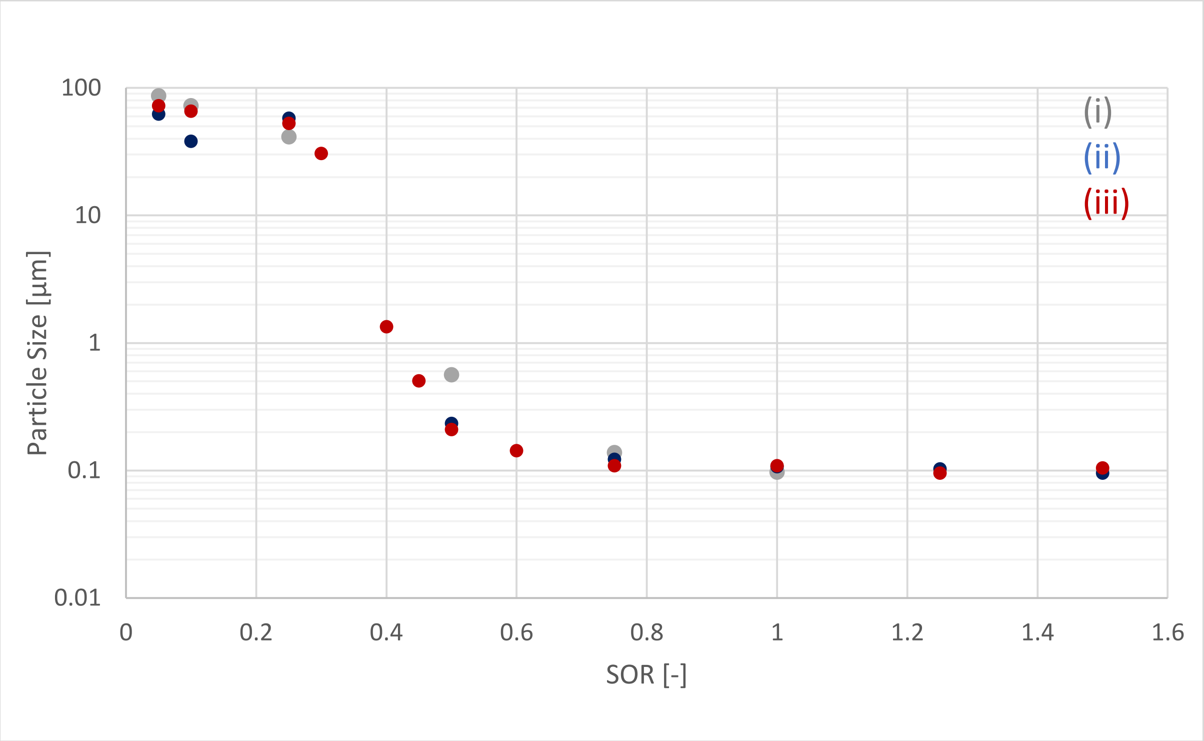

Figure 2: Sauter mean diameter in respect to the surfactant-to-oil ratio using a single-contact microfluidic mixer with (i) without additional heating at the pump inlet/pump, (ii) with additional heating at the pump inlet/pump, and (iii) with additional heating at the pump inlet/pump and additional 10 min of mixing after sample collection.
